# Neutrophil to Lymphocyte Ratio as a Prognostic Marker in Amyotrophic Lateral Sclerosis

**DOI:** 10.3390/biom13121689

**Published:** 2023-11-23

**Authors:** Camille Cotet, Hugo Alarcan, Olivier Hérault, Philippe Corcia, Patrick Vourc’h, Christian R. Andres, Hélène Blasco, Charlotte Veyrat-Durebex

**Affiliations:** 1 Laboratoire de Biochimie et Biologie Moléculaire, CHRU Bretonneau, 2 Boulevard Tonnellé, 37000 Tours, Francehugo.alarcan@univ-tours.fr (H.A.); patrick.vourch@univ-tours.fr (P.V.); christian.andres@univ-tours.fr (C.R.A.); helene.blasco@univ-tours.fr (H.B.); 2 UMR 1253 iBrain, Université de Tours, Inserm, 10 Boulevard Tonnellé, 37000 Tours, France; philippe.corcia@univ-tours.fr; 3 Service d’Hématologie Biologique, CHRU Bretonneau, 2 Boulevard Tonnellé, 37000 Tours, France; olivier.herault@univ-tours.fr; 4 Service de Neurologie, CHRU Bretonneau, 2 Boulevard Tonnellé, 37000 Tours, France

**Keywords:** amyotrophic lateral sclerosis, neutrophils, lymphocytes, NLR, survival, prognosis

## Abstract

Amyotrophic lateral sclerosis (ALS) is the most common neurodegenerative motor neuron disease and remains misunderstood with a difficult diagnosis and prognosis. The implication of the immune system is recognized in ALS pathophysiology, hence the interest in leucocyte count as lymphocytes and neutrophils. The neutrophil-to-lymphocyte ratio (NLR) has recently been used as a prognosis factor to assess the progression of ALS. Thus, the aim of this study was to analyze the evolution of the NLR during disease evolution in a French cohort of ALS patients and its relation with survival. In this monocentric retrospective study, clinical parameters and NLR were collected in ALS patients followed at the University Hospital of Tours (France). ALS patients were subdivided into three groups regarding their NLR value at inclusion: group 1 (NLR < 2); group 2 (NLR: 2–3); group 3 (NLR > 3). A comparison of qualitative and quantitative clinical and biological variables between NLR groups was performed. Then, Cox regressions were carried out to determine the association of NLR with survival. We observed a significant correlation of NLR with ALSFRS-r score (*p* < 0.0001) and with vital forced capacity (*p* = 0.0004) at inclusion. We observed that increased NLR at diagnosis is associated with decreased ALS patients’ survival.

## 1. Introduction

Amyotrophic Lateral Sclerosis (ALS), the most common motor neuron neurodegenerative disease, is heterogeneous. The onset can be bulbar, spinal, or more rarely respiratory. Patients’ follow-up is a real challenge since there are no diagnostic and prognostic biomarkers. The mean survival is 3 to 5 years after diagnosis but a non-negligible fraction of patients has a long survival time in contrast with another fraction with very rapid evolution to death, so the importance of a predictive marker of disease evolution is crucial. Usually, in France, patients are followed every 3 months with a clinical checkup where usual clinical parameters are collected: Body Mass Index (BMI), Amyotrophic Lateral Sclerosis Functional Rating Scale-revised (ALSFRS-r) score, and Forced Vital Capacity (FVC) with a spirometry exam. Blood tests are also performed. No blood marker has been validated for ALS prognosis for now except plasma neurofilament light chain (NfL) concentrations. Several recent studies showed that NfL increase was associated with a higher ALS risk [1], thus showing its interest as a diagnostic and prognostic marker [2]. Routine parameters used to follow ALS evolution are not relevant enough to be used for predictive purposes, and even NfL is not routinely used in France to modify patients’ care according to the results.

The pathophysiology of the disease is still poorly understood and includes many mechanisms: aggregation and accumulation of ubiquitinated proteins in motor neurons, alterations in the RNA metabolism, excitotoxicity of glutamate, oxidative stress, mitochondrial dysfunction, or neuroinflammation [3]. Interestingly, some authors reported a link between systemic immunologic activation and ALS progression [4,5] and some studies revealed that monocyte and neutrophil blood count were associated with bulbar symptoms, disease severity, rate of disease progression, and survival respiratory impairment [6].

According to these recent findings, interest in the Neutrophil Lymphocyte Ratio (NLR) recently emerged in ALS since Italian and Chinese retrospective studies showed a significant link between NLR and disease progression and/or survival [7,8,9].

We aimed to analyze NLR correlation to survival in our French cohort of ALS patients using baseline NLR (at diagnosis) but also at several times in the disease evolution, since knowledge of the evolution of this parameter in ALS is essential to consider it as a prognostic marker. To our knowledge, it is the first time that NLR relation with ALS prognosis was studied in a longitudinal manner, and this parameter has never been analyzed in a French cohort.

## 2. Materials and Methods

### 2.1. Study Design

In this retrospective study, 906 ALS patients were followed at the University Hospital of Tours (France) between June 2008 and April 2022. The diagnosis was set up using the revised El Escorial criteria [10]. Patients were followed up at an interval of 3 months from the date of registration. Several clinical information were collected: the date of initial symptoms, age, sex, site of onset, weight and Body Mass Index (BMI) at diagnosis, forced vital capacity (FVC), and ALSFRS-r score at the diagnosis consultation in our ALS center and for the following 12 months. We included patients having an ALSFRS-r score and a blood test with NLR within 45 days from the diagnosis consultation at the ALS Center of Tours. We excluded patients with hematologic or infectious diseases such as leukemia or pneumonia. Patients with an onset site other than spinal or bulbar were excluded. 

The duration of the disease corresponds to the period of time between the date of the first symptoms and the date of death. Indeed, we considered the time of survival from symptom onset to death for multivariable continuous Cox regression [8]. The evolution of parameters from the inclusion to 6 or 12 months was determined as the percentage of variation of weight, FVC, and ALSFRS-r score.

A blood test corresponds to a blood cell count test using flow cytometry on venous ethylenediaminetetraacetic acid (EDTA) samples. Hematologic parameters were collected at each consultation (3 months, 6 months, 9 months, 12 months): white blood cell (monocytes, basophils, neutrophils, eosinophils, and lymphocytes) count, red blood cell count, hemoglobin, platelet count, hematocrit, mean corpuscular volume, mean corpuscular hemoglobin, NLR and neutrophils-to-monocytes ratio (NMR). NLR corresponds to the ratio of the neutrophil count to the lymphocyte count while NMR was calculated as the ratio of the neutrophil count to the monocyte count.

### 2.2. Statistical Analysis

According to previous studies [7], ALS patients were subdivided into three groups according to the NLR: value group 1 (NLR < 2); group 2 (NLR: 2–3); group 3 (NLR > 3).

Comparisons of qualitative and quantitative variables, corresponding to patients’ biological and clinical diagnostic data, between NLR groups were performed using respectively χ2 and Kruskal–Wallis tests to highlight potential subpopulations in our cohort. *p*-value thresholds were adjusted for multiple comparisons by the Bonferroni method. When the Kruskal–Wallis test was significant, a Wilcoxon test for each pair was performed.

Survival for any group was determined using the Univariate Cox proportional model. Kaplan–Meier curves were defined to illustrate survival. Times with significant *p*-values in a univariate model were analyzed in a multivariate Cox proportional hazards model adjusted for onset age, onset site, and clinical parameters FVC, ALSFRS-r, and BMI at inclusion, to determine hazard ratios (HR) and their 95% Confidence Intervals (CI). This analysis used a continuous and categorized NLR.

Statistics analysis was performed using JMP^®^ statistical software version 10.0.0 (SAS InstituteInc., Cary, NC, USA).

## 3. Results

### 3.1. Cohort’s Characteristics

Of 906 patients followed in the studied period, only 359 had hematologic parameters assessment and were included. A comparison of clinical parameters was conducted between patients with and without assessment of hematologic parameters at diagnosis and did not reveal significant differences, allowing us to proceed without any selection bias. Bulbar onset site concerned 33.4% of ALS patients while 66.6% had spinal form, and 48% of our cohort was female patients. The mean age at diagnosis was 67.5 years, the mean reference of weight was 67 kg, the mean FVC was 71.3%, the mean ALSFRS-r was 34.5, the mean BMI was 24.3 kg/m^2^ for the cohort, and the mean NLR was 3.05 at the inclusion.

### 3.2. Correlation of Blood Cell Count with Parameters of Disease Progression at Diagnosis

We have evaluated the correlation of various blood cell counts (red blood cells, total leucocytes, neutrophils, eosinophils, basophils, lymphocytes, monocytes, and platelets), as well as cell ratios (neutrophil-to-lymphocyte ratio NLR and neutrophil to monocyte ratio NMR), with parameters of disease progression, i.e., forced vital capacity (FVC) and ALSFRS-r score. We observed a correlation of neutrophil, eosinophil, and monocyte counts with FVC (r = −0.166 with *p* = 0.0040, r = 0.2434 with *p* < 0.0001, and r = −0.1745 with *p* = 0.0025, respectively), a positive correlation of lymphocytes with FVC and ALSFRS-r score (r = 0.1758 with *p* = 0.0023 and r = 0.1736 with *p* = 0.0012, respectively), and a negative correlation of NLR with both FVC and ALSFRS-r score (r = −0.2026 with *p* = 0.0004 and r = −0.2266 with *p* < 0.0001, respectively).

### 3.3. NLR as a Relevant Parameter to Understanding Pathophysiology

**Figure 1:**
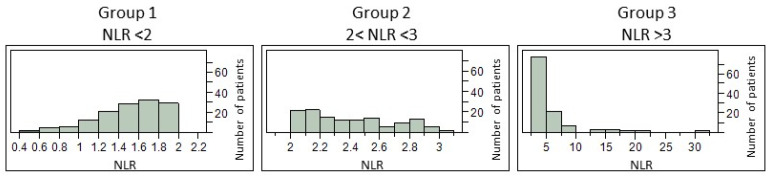
Distribution of NLR values within each group.

Focusing our study, especially on neutrophil/lymphocyte ratio (NLR), we distributed patients in 3 groups depending on their NLR at diagnosis: group 1 with baseline NLR values < 2 (n = 127); group 2 with NLR values [2,3] (n = 122); and group 3 with NLR values > 3 (n = 110). The distribution of NLR values within each group is presented in Figure 1. A comparison of their demographic and clinical characteristics is detailed in Table 1. We highlighted a significant difference in the ALSFRS-r score between each group (*p* = 0.0010). A deeper comparison using the Wilcoxon test for each pair of groups revealed a significant difference between groups 1 and 2 vs. group 3 (*p* = 0.0019 and *p* = 0.0008, respectively) and no significant difference between groups 1 and 2 (*p* = 0.5379). As expected, significant differences between the three groups were found for white blood cells (neutrophils, eosinophils, lymphocytes, and monocytes), NLR, and NMR. Patients of Group 3 had significantly higher levels of leucocytes, neutrophils, NLR, and NMR, but lower levels of eosinophils, basophils, lymphocytes, and monocytes (Table 1). For the rest of the analyses, the 3 groups were compared separately.

### 3.4. NLR as a Performant Parameter to Help in ALS Prognostic

As mentioned above, three blood test parameters were correlated with ALSFRS-r score at inclusion (neutrophils; lymphocytes; NLR). Neutrophil count and NLR were also found to be correlated to FVC at diagnosis. However, correlation of these three parameters with ALSFRS-r or FVC was not found at other time points (three, six, and twelve months), only neutrophil count was found to be correlated with ALSFRS-r and FVC at three months (*p* < 0.0001, n = 115) and NLR with ALSFRS-r (*p* = 0.0008, n = 144) at three months follow-up.

**Figure 2:**
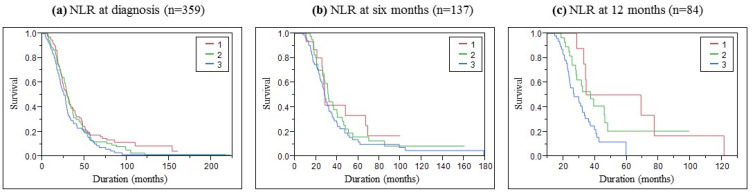
Survival Kaplan–Meyer curves of ALS patients according to the NLR (**a**) at inclusion, (**b**) 6 months, and (**c**) 12 months follow-up.

Kaplan–Meier analysis presented in Figure 2 showed that the survival time was different among the three NLR groups. We performed pairwise log-rank tests to evaluate the difference between groups. At inclusion, close but significantly different curves were found between groups 1 and 3 (*p* = 0.0399). The pairwise test at six months did not highlight a significant difference, but we can see a trend of better survival in group 1 with lower NLR values. After one year, we observed that group 3 with NLR > 3 quickly decreased while group 1 stepping decreased, and group 2 stayed between. This observation was confirmed by a significant difference between the curves of groups 1 and 3 (*p* = 0.0126) and between groups 2 and 3 (*p* = 0.0342). Survival medians according to the NLR group were presented in Table 2.

As presented in Table 3, univariate continuous Cox analysis allows for studying the early NLR variation impact on survival. Increased NLR at each time follow-up significantly increased the probability of decreased survival (at inclusion: *p* = 0.0158; 6 months: *p* = 0.0062; 12 months: *p* = 0.0006) with an increasing hazard ratio with disease progression (at inclusion: HR = 1.066; 6 months: HR = 1.131; 12 months: HR = 1.172) (Table 3). In multivariate analysis, NLR was significantly correlated with survival only at 6 and 12 months.

The multivariate Cox proportional hazards model was adjusted for onset age, onset site, and clinical parameters FVC, ALSFRS-r, and BMI collected at the patient’s inclusion. We could not adjust with other laboratory data because of the strong correlation between NLR and complete blood count parameters.

## 4. Discussion

Immune dysregulation in ALS is unclear but the known consequences are enhanced neuroinflammation, impaired motor neuron function, and fast disease progression [5]. For that reason, it may be important to understand this dysregulation to be able to evaluate prognosis and treat patients in consequence. Correlation between neutrophil blood count and clinical parameters [6] or survival [11] had already been observed. Indeed, monocyte and neutrophil blood counts were associated with bulbar symptoms, disease severity, rate of disease progression, and respiratory impairment [6]. Also, a higher neutrophil count early in ALS was associated with shorter survival, particularly for female patients [11]. The present study was carried out to evaluate the interest of complete blood cell count, especially baseline and following NLR values, for prognosis in ALS patients. We performed our analyses on a large cohort with an extensive set of hematologic laboratory data to improve our sensitivity.

Our results have shown that a higher NLR value at diagnosis or during disease follow-up constituted an increased risk factor of shorter survival which is in line with the studies cited. Indeed, higher NLR is associated with a faster degradation of the clinical parameters and shorter survival in ALS patients. The predictive value of NfL in ALS was found to be useful in the 5-year post-diagnosis samples, but no correlations remained significant after adjustment for multiple comparisons [1]. Thus, given these results, NLR could be a complementary or full-fledged solution depending on the patient. The NLR is a reliable and standardized hematological parameter. Wei et al. observed a significant correlation between NLR and survival in ALS patients [7], higher NLR values at diagnosis were associated with poorer survival outcomes. Several studies worked by dividing their patients into groups depending on their NLR values, according to a threshold or tercile [7,8]. Another research had the same conclusion and used terciles as well but took more parameters into consideration in multivariate analysis, such as protein reactive C [12]. Grassano et al. also showed an increased neutrophils and a higher NLR are associated with reduced survival and a faster progression of disease [9]. NLR value at diagnosis could be useful for prognosis with a premature variation of the NLR values in the first months of evolution. Therefore, NLR could be a predictive marker of disease evolution in ALS patients.

### 4.1. Correlation between NLR and Clinical Parameters

Our study demonstrated a negative correlation between neutrophil count and NLR with ALSFRS-r and FVC at diagnosis. Indeed, a previous study observed a correlation between markers of poor prognosis and increased neutrophil count and NLR [9]. A previous study showed that CD16 neutrophil count was correlated with disease severity, reflected by the ALSFRS-r score, and rate of progression (ALSFRS-r score evolution) [6]. The rate of disease progression was positively correlated to NLR in a recent multicenter investigation that included 146 patients [8]. They only analyzed the correlation of NLR at inclusion, while we integrated more parameters that were correlated with ALSFRS-r score: neutrophil count and lymphocyte count, according to NLR, at several steps of disease progression. NLR cut-offs were already defined in several diseases as coronary artery disease [13] or cancer [14]. Groups were defined according to the biological pertinence of this parameter: NLR was generally accepted as a normal range with values between 1–2, while NLR over 3 indicates a pathological state [15]. According to Wei et al. results, we observed a significant difference between ALSFRS-r scores in patients separated according to their NLR [7].

### 4.2. NLR as a Risk Factor for Poor Survival

The univariate continuous Cox model showed that NLR was significantly associated with survival at inclusion, 6, and 12 months. Leone et al. explored the continuous univariate association of NLR with survival only at inclusion and found a significant correlation [8]. Thus, we confirmed previous studies by highlighting that higher NLR values were significantly associated with survival in unadjusted models [8,9,12]. The multivariate Cox proportional hazards model with continuous variable was significantly associated with survival for 6 and 12 months, despite the loss of patients with NLR and clinical parameters during follow-up. Our adjusted models were consistent with Leone et al. and Wei et al. results [7,8].

Kaplan–Meier curves showed that survival was associated with NLR values. This confirmed the results observed by Leone et al. highlighting a significant association between categorized NLR and survival at inclusion, for groups with higher NLR values (NLR > 2.326) [8]. Lastly, another study that evaluated the interest of NLR in ALS patients also showed a significant correlation between NLR and survival for groups with NLR values > 3 [7]. These studies found that higher NLR values were a risk factor in multivariate models while we noted the same conclusion in univariate models and a trend in this way in multivariate ones. The exploration of more covariables in the multivariate Cox model would be interesting, such as ALSFRS-r slope during follow-up, clinical parameters variation during follow-up or other biological parameters. Differences observed between our results and results of Korean and Chinese investigations could be explained by the fact that they included more parameters in their multivariate analysis like CRP, uric acid, albumin, and glycated hemoglobin. Also, drug-related parameters were not analyzed in our study. It would also be interesting to use a statistical approach using joint models in such a longitudinal study due to the significant loss of data during patient follow-up.

Our results showed an association between NLR and survival, and thus with prognosis for ALS patients. These results are consistent with the pathophysiology of the disease in which neuroinflammation, including activation of the immune system cells such as neutrophils and lymphocytes, plays an active role [4].

## 5. Conclusions

A significant correlation between baseline NLR and CVF or ALSFRS-r score was demonstrated in ALS patients. Furthermore, increased NLR is negatively associated with ALS patients’ survival. We observed that the hazard ratio in the categorized NLR model showed that the risk increased with NLR values at each disease’s follow-up, with an increasing hazard ratio over the months.

This longitudinal analysis brought new information never collected before. Our results confirm the interest in using analysis of NLR values as biomarkers to assist in the prognosis of ALS patients eventually in addition to NfL concentrations.

## Figures and Tables

**Table 1:** Comparisons of ALS patients’ diagnostic data among the three subgroups according to NLR at diagnostic.

Patients with NLR = 359	Group 1 n *=* 127	Group 2 n *=* 122	Group 3 n *=* 110	*p*-Value
Age diagnostic (years)	65.9 ± 11.9	67.3 ± 10.6	68.9 ± 11.6	0.1288
Weight (kg)	66.7 ± 15.1	69.3 ± 14.8	64.5 ± 14.2	0.0474
Sex (%)	Female: 59.1	Female: 48.4	Female: 44.5	0.0649
	Male: 40.1	Male: 51.6	Male: 55.5	
Onset site (%)	Bulbar: 34.6	Bulbar: 38.0	Bulbar: 30.9	0.5258
	Spinal: 65.4	Spinal: 62.0	Spinal: 69.1	
Duration (month)	34.5 ± 27.8	34.0 ± 28.1	28.5 ± 26	0.0281
FVC (%)	93.8 ± 27.1	91.1 ± 26.7	80.8 ± 30.3	0.0098
ALSFRS	38.2 ± 5.4	38.1 ± 6.5	35.1 ± 7.6	0.0010 *
BMI kg/m^2^	24.6 ± 4.9	24.9 ± 4.4	23.5 ± 4.5	0.0485
PNN (G/L)	3.2 ± 0.9	4.11 ± 1.1	6.0 ± 2.4	<0.0001 *
Hemoglobin (g/L)	139.2 ± 12.5	141.1 ± 12.0	140.9 ± 14.1	0.3510
Red blood cells (T/L)	4.5 ± 0.4	4.6 ± 0.4	4.6 ± 0.5	0.1313
Hematocrit (%)	41.5 ± 3.7	42.0 ± 3.5	42.2 ± 4.3	0.2548
MCV (fL)	92.1 ± 4.2	91.3 ± 3.9	92.1 ± 4.1	0.1275
MCHC (g/dL)	33.6 ± 0.8	33.5 ± 0.7	33.4 ± 0.8	0.1536
MCH (pg)	30.9 ± 1.6	30.7 ± 1.5	30.8 ± 1.6	0.2311
PMV (fL)	8.9 ± 1.0	9.0 ± 0.9	8.9 ± 1.1	0.5314
Leucocytes (G/L)	6.0 ± 1.5	6.5 ± 1.6	8.1 ± 2.6	<0.0001 *
PNE (G/L)	0.18 ± 0.12	0.15 ± 0.11	0.12 ± 0.09	0.0004 *
PNB (G/L)	0.041 ± 0.022	0.037 ± 0.019	0.036 ± 0.029	0.0120
Lymphocytes (G/L)	2.1 ± 0.6	1.7 ± 0.4	1.3 ± 0.4	<0.0001 *
Monocytes (G/L)	0.49 ± 0.15	0.53 ± 0.18	0.63 ± 0.21	<0.0001 *
Platelets (G/L)	234.8 ± 58.7	235.7 ± 66.3	254.4 ± 70.9	0.0193
NMR	6.9 ± 2.4	8.2 ± 2.3	10.1 ± 3.4	<0.0001 *

* Significant after Bonferroni correction; FVC: forced vital capacity; ALSFRS: Amyotrophic Lateral Sclerosis Functional Rating Scale; BMI: Body Mass Index; PNN: neutrophils polynuclear; MCV: Mean Cell Volume; MCHC: mean cell hemoglobin concentration; MCH: Mean Cell Hemoglobin; PMV: Platelets Mean Volume; PNE: eosinophils polynuclear; PNB: basophils polynuclear; NLR: Neutrophil–Lymphocyte Ratio; NMR: Neutrophil-to-Monocyte Ratio. Quantitative values are reported as mean +/− standard deviation.

**Table 2:** Survival medians according to NLR group at inclusion, 6 and 12 months follow-up.

Survival	Median in Months (Confidence Interval at 95%)		
	At Inclusion	At 6 Months	At 12 Months
Group 1 (NLR < 2)	29 (24.3–34.3)	28.2 (21–67)	51.75 (28.6–121.3)
Group 2 (NLR 2–3)	30 (26.9–33.8)	31.9 (27.7–40.2)	37.3 (27.8–46.2)
Group 3 (NLR < 3)	25.7 (21.5–29.1)	28 (24.6–32.1)	26.9 (23.6–33.4)

**Table 3:** Univariate and multivariate Cox regression analyses according to continuous NLR. * Significant *p*-value < 0.05.

	Univariate		Multivariate	
	Hazard Ratio (95% CI)	*p*-Value	Hazard Ratio (95% CI)	*p*-Value
NLR_M0	1.066 (1.013–1.111)	0.0158 *	1.057 (0.954–1.159)	0.2764
NLR_M6	1.131 (1.038–1.215)	0.0062 *	1.258 (1.036–1.510)	0.0213 *
NLR_M12	1.172 (1.075–1.269)	0.0006 *	1.368 (1.022–1.829)	0.0358 *

## Data Availability

Data are available at CHRU de Tours.
